# Fracture risk in long term care: a systematic review and meta-analysis of prospective observational studies

**DOI:** 10.1186/1471-2318-14-130

**Published:** 2014-12-03

**Authors:** Rasha Khatib, Nancy Santesso, Laura Pickard, Osman Osman, Lora Giangregorio, Carly Skidmore, Alexandra Papaioannou

**Affiliations:** Clinical Epidemiology and Biostatistics, McMaster University, 1280 Main Street West, Hamilton, ON L8S 4 K1 Canada; Population Health Research Institute, Hamilton Health Sciences and McMaster University, 237 Barton Street East, Hamilton, ON L8L 2X2 Canada; Department of Medicine, Faculty of Health Sciences, McMaster University, 1280 Main Street West, Hamilton, ON L8S 4 K1 Canada; Hamilton Health Sciences- St. Peter’s Hospital, 88 Maplewood Avenue, Hamilton, ON L8M 1 W9 Canada; Department of Public Health and Health Systems, University of Waterloo, 200 University Avenue West, Waterloo, ON N2L 3G1 Canada; Department of Kinesiology, University of Waterloo, 200 University Avenue West, Waterloo, ON N2L 3G1 Canada

**Keywords:** Fractures, Long term care, FRAX, Risk assessment

## Abstract

**Background:**

The risk factors associated with fractures have been well-characterized in community dwelling populations, but have not been clearly defined in long-term care (LTC) settings. The objective of this review was to identify risk factors for fractures in LTC settings.

**Methods:**

We searched MEDLINE, the Cochrane Library, EMBASE and CINAHL up to June 2014, scanned reference lists of articles and consulted with experts in the field to identify relevant prospective cohort studies that evaluated risk factors associated with fracture incidence in LTC. We included studies that assessed the association between risk factors included in the WHO-Fracture Risk Assessment Tool (FRAX®) or other predictors relevant to LTC (psychotropic medications, cognitive impairment, mobility, and falls). All articles were screened and extracted by two authors. Available data on the association between a given risk factor and fracture incidence were pooled when possible. We used the GRADE criteria to provide a summary of evidence. The GRADE approach defines the quality of a body of evidence as the extent to which one can be confident that an estimate of effect or association is close to the quantity of specific interest.

**Results:**

We identified 13 prospective cohort studies which examined fracture incidence among LTC residents. Most predictors showed moderate increases in fracture risk, but the quality of the evidence was often low. Moderate quality evidence showed that prior fractures and falls may moderately increase the risk of fractures. Being a woman and cognitive impairment are probably associated with a small increase. The effect of mobility and psychotropic medication use is still uncertain primarily due to the various definitions used in the studies and difficulty summarising the results.

**Conclusions:**

In addition to criteria used in the FRAX assessment tool, such as a previous fracture and female gender, we found that falls and cognitive impairment are also associated with a small to moderate increases in the risk of fractures in LTC. Developing an assessment tool that includes risk factors that are specific to LTC may improve the identification of individuals who can benefit from fracture prevention programs in these settings.

## Background

Fractures of the hip and spine are responsible for loss of mobility [1], death [2] and increased health care costs [3, 4]. Over one quarter of hip fractures occur in long-term care (LTC) residents [5, 6]. Individuals who live in LTC homes are two to four times more likely to have a fracture than their community dwelling age matched peers [5, 6]. This may be due in part to the prevalence of osteoporosis (80-85%) [7] and the high incidence of falls. Hip fractures, one of the most serious fall related injuries, are one of the leading causes of hospitalization for LTC residents [6] and the pain and delirium resulting from the fracture can be distressing for residents and their families. Vertebral fractures have been documented in 30% of residents [8] and have been associated with a significant source of pain [9–11], anxiety, depression [10, 11], disability [9], and impaired pulmonary function [10].

Treatments to reduce the risk of fractures are available [12]. A number of risk assessment tools have been developed to identify individuals at risk of fractures who could benefit from these treatments. Many of these tools were developed by identifying risk factors for fractures in large epidemiological studies of community dwelling older adults. The Fracture Risk Assessment Tool (FRAX®) has been validated in a number of countries and risk can be calculated with and without bone mineral density (BMD) [13].

Other assessment tools that have been validated for assessing fracture risk among community dwelling older adults include the CAROC (Canadian Association of Radiologists and Osteoporosis Canada Risk assessment Tool) [14], the Simple Calculated Risk Estimation Score (SCORE) [15], FRACTURE index (SOF) [16] and Qfracture [17, 18].

However, there is limited data regarding the validity of these tools when estimating fracture risk among residents of LTC homes. Many of the tools require BMD measurements, which can be challenging for LTC residents to access given their limited mobility and the difficulty they may have positioning for scans. Tools such as CAROC and FRAX which estimate 10 year fracture risk may not be appropriate in LTC homes where 40% of residents may die within three years [19] and which do not include other risk factors for fracture which may be applicable to LTC settings, such as falls.

Some studies attempted to assess the applicability of these tools in LTC homes. Greenspan and colleagues [20] explored the implications of employing FRAX with BMD, FRAX without BMD, and other imaging techniques to estimate fracture risk among nursing home residents. FRAX without BMD has been proposed for use when results of imaging tests are not readily available. When this method was applied, 98% of residents were considered candidates for treatment. No one method of assessing fracture risk appeared to be optimal.

We systematically reviewed the literature to identify risk factors for incident fractures that have been reported in prospective cohort studies conducted among residents in LTC settings. We have focused on evaluating the evidence based on risk factors that are components of the FRAX tool which is utilized internationally. We also included factors which may be pertinent to fracture risk in LTC settings but were not included in the FRAX tool.

## Methods

### Search strategy, eligibility criteria, and study selection

We searched MEDLINE (1946 to June 2014), Cochrane Library (up to May2014) including CENTRAL, DARE, and CDSR (Cochrane Database of Systematic Reviews), EMBASE (1947 to June 2014), and CINAHL (1981 to June 2014); scanned reference lists of articles; used the related articles search in PubMed using two relevant articles [21, 22] and, consulted with experts in the field. An experienced Cochrane librarian developed a systematic search strategy using subject headings and keywords such as ‘long-term care’, ‘risk prediction’ and ‘fractures’. No limits were applied to language or study type (Appendix 1). Nursing homes, care homes, residential care facilities, skilled nursing facilities and retirement homes were defined as LTC facilities in this paper. Articles were screened independently by two reviewers according to the eligibility criteria listed below.

#### Eligibility criteria of included studies

*Population:* Studies that included male and female participants ages 50 and above and living in a long term care (LTC) facility were considered. LTC facilities included nursing homes, care homes, residential care facilities, skilled nursing facilities, and retirement homes.

*Exposures:* 1. FRAX predictors (Age, gender, BMI, prior fractures, glucocorticoid use, bone mineral density, rheumatoid arthritis, parental fractured hip, current smoking, secondary osteoporosis and alcohol intake). 2. Other predictors that have not been assessed in FRAX but that may be relevant in long term setting (psychotropic medications, cognitive impairment, mobility, and falls).

*Outcome measures:* any incident fracture.

*Types of studies:* Prospective cohort studies.

*Exclusions*: no exclusions were made based on language or date of publication.

### Data collection

We extracted data following a pilot tested data extraction form including study characteristics (country, setting, time to follow up, sample size, and predictors), population characteristics (age, percent females, inclusion criteria), and outcome measures (type of fracture). Study authors were contacted when information on the exposure measure were unclear. Data was extracted by one author and verified by another. A third author resolved disagreements.

We collected data on risk factors included in the FRAX model (age, gender, body mass index (BMI), prior fractures, glucocorticoid use, bone mineral density (BMD), rheumatoid arthritis, parental fractured hip, current smoking, secondary osteoporosis and alcohol intake [23]. Four additional risk factors for fractures identified a priori due to relevance in LTC settings and included psychotropic medications, cognitive impairment, mobility and falls.

### Synthesis of results and assessment of risk of bias

The risk associated with fractures was pooled across studies when possible. When studies expressed the risk associated with fractures using different risk statistics (e.g. Risk Ratio (RR), Odds Ratio (OR), and Hazard Ratio (HR)), we converted HR to RR and assumed OR provided a good estimate of RR [24]. Most of the included studies did not provide raw data for the number of fractures. Therefore, we used the inverse variance method to pool these estimates using Review Manager 5 [25].

A random effects model was used to pool the effect estimates. Heterogeneity across pooled studies was expressed using the I^2^ statistic, which describes the percentage of the variability in effect estimates that is due to heterogeneity in meta-analyses. Heterogeneity was assessed as not important (0 to 40%), moderate (30 to 60%), substantial (50 to 90%) or considerable (75 to 100%) [26]. We conducted post hoc sensitivity analyses to explore moderate heterogeneity or greater. Pooled proportions and pooled effect measures were presented using forest plots. A narrative description of studies that developed a risk prediction tools based on the risk factors identified in each study is also presented.

We used the Guidelines for Assessing Quality in Prognostic Studies on the Basis of Framework of Potential Biases to assess bias in each study [27]. The following criteria were assessed: (1) representation of sample; (2) study attrition; (3) prognostic factor measurement; (4) outcome measurement; (5) confounding measurement and account; and, (6) appropriateness of statistical analysis.

### Quality of evidence

The GRADE approach was used to assess the quality of the evidence and confidence in the effects [28]. Although guidance for prognostic studies has not been developed formally for prognostic reviews, we assessed the results for each risk factor according to the GRADE criteria (risk of bias, imprecision, inconsistency, indirectness, publication bias, magnitude of effect, plausible confounding effects, and dose response). The quality of the evidence was assessed as high, moderate, low or very low, with evidence from prospective cohort studies starting as high quality and downgraded accordingly. High quality evidence indicates that we are very confident that the true effect lies close to that of the estimate of the effect; and very low that we have very little confidence in the effect estimate and the true effect is likely to be substantially different from the estimate of effect.

We assessed the strength of the association between each risk factor and fractures using the Monson criteria [29] suggested by Oleckno [30]. A risk ratio ranging between 1.1-1.5 was considered weak/small, 1.6-3.0 was considered moderate, and an association of 3.1 or higher was considered strong/large. When the confidence intervals were wide and/or included no effect and the potential for small, moderate or large effects, the effect was uncertain.

## Results

### Characteristics of included studies

**Figure 1:**
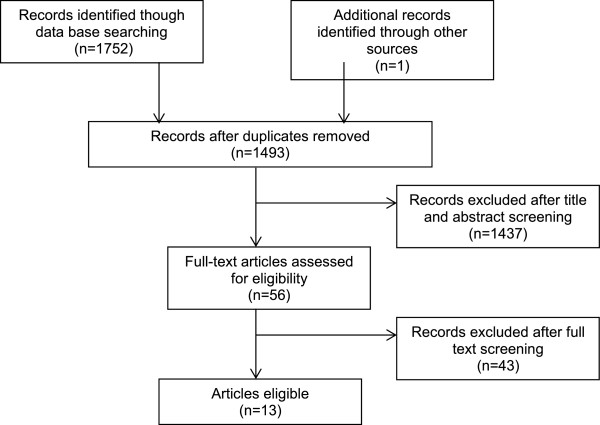
**PRISMA flow diagram.**

Our search identified 1,493 unique citations and 13 studies met the inclusion criteria (Figure 1). Results from two studies [21, 31] could not be pooled and their characteristics are presented separately at the end of the results section. Table 1 provides details of the characteristics of the 11 studies that were included in the meta-analyses. All studies had a prospective cohort design, were published in English, and included 153,563 LTC residents. Sample size varied between 184 [32] and 83,959 [33]. Three studies recruited data from only one LTC facility [32, 34, 35] and three studies collected data from multiple homes [8, 21, 36–38]. The remaining studies used the Fracture Risk Epidemiology in the Frail Elderly (FREE) dataset [22, 31], provincial administrative data [39] or the Minimum Data Set (MDS) [33, 40, 41]. The majority of studies retained did not apply any exclusion criteria to their population of interest for their analysis. However, five studies were more selective and only included patients with a prior fracture [32], patients who could walk independently [34, 36], female residents [36, 38], and excluded bed-bound residents [22]. Mean age of residents was more than 80 years old in all included studies.

**Table 1:** **Characteristics of included studies**

Author	Country	Setting	Age, mean (μ) ± SD	% females	Sample size	Follow-up	Outcome
Berry, 2008 [32]**	USA	1 LTC	μ: 89.0 (±6.1)	79%	184	8.5 years	Osteoporotic fractures
Broe, 2000 [35]	USA	1 LTC	μ: 88.0 (±6.2)	74%	252	6.6 years	Osteoporotic fractures
Chandler, 2000 [38]**	USA	47 NH	μ: 85.0 (±7.0)	100%	1427	18 months	Osteoporotic fractures
Chen, 2009 [22]**	Australia	NH, ICF	μ: 85.6	75%	1,894	4 years	Hip fracture
		Free dataset					
Colon-Emeric, 2003 [40]	USA	NH	Fracture group μ: 81.96 ± 0.19	Fracture group 79%	13,516	1-2 years	Hip fracture
		MDS dataset					
				No fracture group: 68%			
			No fracture group μ: 80.83 ± 0.05				
Dobnig, 2007 [36]	Austria	NH	Ctrl μ: 83.8 (±6.2), Hip fracture μ: 83.6 (±6.3), Non-vertebral. Fracture μ 83.8 (±6.3)	100%	1,664	2 years	Hip* and Non- vertebral fracture
		95 homes					
Huybrechts, 2011 [39]	Canada	NH	Atypical antipsychotics μ: 84.0 (±6.6)	Atypical antipsychotics: 58%	10,900	180 days	Femoral Fracture
		Data from BC Ministry of Health					
			Conventional antipsychotics μ: 83.0 (±6.8)	Conventional antipsychotics: 55%			
Huybrechts, 2012 [33]	USA	NH	Conventional antipsychotics μ: 83.3	Conventional APMs: 69% atypical AMPS: 75%	83,959	180 days	Hip fracture
		MDS, Medicaid, OSCAR					
			Atypical antipsychotics: 83.3				
Lyles, 2008 [41]	USA	NH	Prior hip fractures μ: 83.3, Other fractures μ: 78.8	Prior hip fractures: 83% Other fractures: 80%	30,665	2 years	Osteoporotic fractures
		MDS and Medicare in North Carolina					
			No prior fracture μ: 80.2	No prior fracture: 63%			
Nakamura, 2010 [37]	Japan	140 NH	Women μ: 85.5 (±7.5), Men μ: 80.3 (±8.6)	76%	8,905	1 year	Hip fracture
Visenten, 1995 [34]**	Italy	1 NH	μ 81.5 (±8.0)	76%	197	3 years	Osteoporotic fractures

LTC = long term care; NH = nursing home; ICF = Intermediate Care Facility; MDS = Minimum Dataset; FREE = Fracture Risk Epidemiology in the Frail Elderly; BC = British Colombia.

*When results for 2 fracture sites were reported, we only included results of hip fractures.

**Study analysis restricted to subpopulation of long term care residents.

Six studies included osteoporotic fractures as their primary outcome [32, 34, 35, 38, 41]. One of the six studies defined osteoporotic fractures according to ICD 9 Codes (820x or 733x for hip fracture and codes 821x-829x for other osteoporotic fractures) [41]. The definition of osteoporotic fractures varied in the remaining five studies and included: subsequent fractures at any skeletal site, excluding toes and hands [32]; at any site, excluding ankle, elbow, finger, or face fractures [35]; radiologically confirmed vertebral fractures occurring after a fall and fractures at any skeletal site excluding skull, toes and fingers, [34]; hip and non vertebral fractures excluding skull, toes and fingers [36]; vertebrae, hip, humerus, wrist, rib, clavicle, pelvis, leg, foot, and toes and excluding face, fingers, ankle and patella [38]. Follow up varied between 180 days [33, 39] and 8.5 years [32].

### Risk of bias and synthesis of results

Overall, the risk of bias of the studies was low. Only one study [32], did not adequately represent the population of interest on key characteristics, sufficient to potentially bias the results. Two studies [33, 40] did not explain how they accounted for loss to follow up. Statistical analysis and adjusting for confounders was appropriate in all 11 studies. Table 2 provides a summary of the evidence and the quality of the evidence for each risk factor in the next section.

**Table 2:** **Summary of effect of FRAX and other predictors of fracture risk in long term care patients**

Risk factor	N studies and participants	Association with fractures	Confidence in effect
**Predictors in FRAX**			
Prior fractures	Pooled: 6 studies; 56,781 participants	Moderate increase in risk	Moderate
		RR = 1.71, 95%CI = 1.30-2.24	⊕ ⊕ ⊕O
Female gender	Pooled: 3 studies; 44,433 participants	Small increase in risk	Moderate
		RR = 1.40, 95%CI = 1.00-1.95	⊕ ⊕ ⊕O
Lower BMI*	Pooled: 2 studies; 1,729 participants	Little to no increase in risk HR = 0.94, 95%CI = 0.90-0.98	low
	Not pooled: 1 study; 128 participants		⊕ ⊕ OO
Older age	Not pooled: 5 studies; 44,745 participants	Small increase in risk	low
			⊕ ⊕ OO
Low BMD	Not pooled: 2 studies; 1,708 participants	Moderate to large increase in risk	low
			⊕ ⊕ OO
Glucocorticoid use	Not pooled: 1 study; 1,550 participants	Moderate to large increase in risk	low
			⊕ ⊕ OO
Rheumatoid arthritis	Not pooled: 1 study; 30,665 participants	Little to no increase in risk	low
			⊕ ⊕ OO
**Predictors not in FRAX**			
Psychotropic medication use	Pooled: 3 studies; 45,962 participants	Uncertain	Very low
	Not pooled: 2 studies; 94,859 participants		⊕OOO
Cognitive impairment	Pooled: 2 studies; 14,773 participants	Small increase in risk	Moderate
	Not pooled: 1 study; 1,894 participants	RR = 1.53, 95%CI = 1.09-2.14	⊕ ⊕ ⊕O
Mobility	Not pooled: 3 studies; 30,132 participants	Uncertain	Very low
			⊕OOO
Falls	Pooled: 4 studies; 44,560 participants	Small to moderate increase in risk	Moderate
		RR = 1.28, 95%CI = 1.04-1.58	⊕ ⊕ ⊕O

*Since both studies included in the meta analysis of BMI reported Hazard Ratios, we did not convert them into relative risks and report pooled results as hazard ratios.

#### 1. Predictors included in FRAX

##### Prior fractures

**Figure 2:**
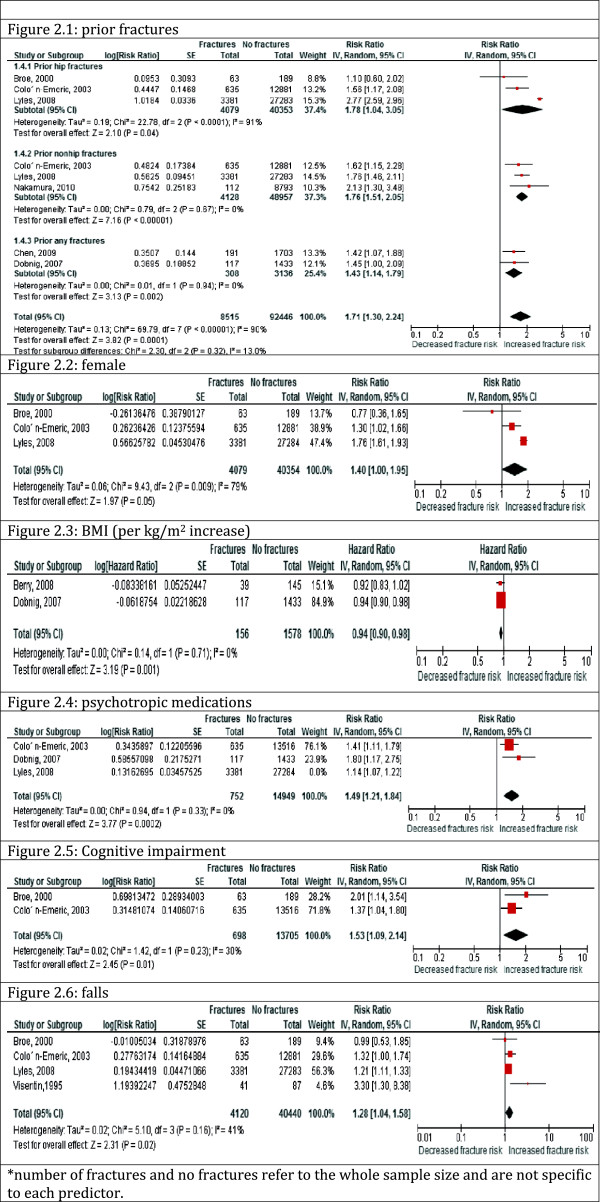
**Pooled effect of each risk factor on future fractures.**

We pooled analyses from six studies (56,781 participants) and found the risk of future fractures associated with prior fracture was RR = 1.71, 95% CI = 1.30-2.24. Heterogeneity was substantial (I^2^ = 90%) and post-hoc exploration was conducted by the site of prior fractures (Figure 2). Pooled risk of future fractures was similar in the presence of a prior hip (RR = 1.78, 95% CI = 1.04-3.05) or prior non-hip fractures (RR = 1.76, 95% CI = 1.51-2.05), but was slightly lower when any prior fracture was used in studies (RR = 1.43, 95% CI = 1.14- 1.79). Prior non-hip fractures were defined as upper or non-hip lower limbs (non-vertebral) in one of the studies [37] and by ICD 9 codes 821–829 in another [41]. The remaining study separated prior fractures into hip fractures and other prior fractures without defining either group more specifically [40, 41]. In those studies reporting on any fracture, prior fractures were captured by interview or chart review and no exclusions by site of fracture were specified [22, 36].

Heterogeneity was low for prior non-hip fractures (I^2^ = 0) and for prior any fractures (I^2^ = 0) but remained high across studies examining the risk associated with prior hip fracture (I^2^ = 91%). Across individual studies, the reported risk of future fracture associated with prior fractures ranged from a small to moderate increase in risk. Two of the studies included selected sub- populations of residents in LTC [22, 36], suggesting indirect evidence. There was therefore, moderate quality evidence for a moderately increased risk of fractures with a history of prior fractures.

##### Female

We pooled three studies (44,433 participants), which assessed the risk of fractures by sex, resulting in RR 1.40, 95% CI = 1.00-1.95 among females when compared to males (Figure 2). Heterogeneity was substantial (I^2^ = 79%) and could not be explained across studies. Reported associations in individual studies ranged from no effect to small effects of female gender. The quality of the evidence was assessed as moderate, supporting a probable small increased risk of fractures in females in comparison to males living in LTC homes.

##### BMI

We pooled hazard ratios from two studies (1,734 participants) without converting to risk ratios, as they both used the same effect measure. The risk of fracture increased with decreasing BMI (HR = 0.94, 95% CI = 0.90-0.98) (Figure 2). There was no heterogeneity across pooled studies (I^2^ = 0). However, the number of events was small suggesting imprecision. A third study categorized BMI using a cut-off at 23 kg/m^2^ and therefore could not be pooled [34]. The study reported a RR 1.30 (95% CI = 0.90-1.80) with BMI lower than 23 kg/m^2^. Berry [32] restricted their analysis to patients with prior fractures only and Visentin [34] restricted their analysis to mobile residents only suggesting indirect evidence. There is low quality evidence that there may be little to no effect of BMI on fracture risk.

##### Age

Five studies reported data on the effect of age on fractures (Table 3). One study categorized age into five year intervals [41]. The remaining four studies assessed the effects of age as a continuous measure. Two of these studies assessed fracture risk per one year increase in age [32, 40], one assessed fracture risk per five years increase in age [35]. The last study assessed fracture risk per increase in one standard deviation of age [34]. Due to variations in the cut off points we were unable to pool effect measures. All five studies suggested small to no association between age and fracture risk. Two studies used selected sub- populations of residents in LTC [32, 34] suggesting indirect evidence. Overall, there is low quality evidence that there may be a small increased risk of fractures at older age.

**Table 3:** **Results of individual studies reporting the effect of age on fracture risk**

Study	Total fractures	Total no fractures	Age group	Effect measure (95% CI)
Broe, 2000 [35]	63	189	Per 5 years	RR = 1.18 (0.91-1.53)
Colon- Emeric, 2003 [40]	635	12,881	Per year	OR = 1.03 (1.01,1.04)
Visentin, 1995 [34]	41	87	Per 1 SD	RR = 1.7 (1.1-2.3)
Berry, 2012 [32]	39	145	Per year	HR = 0.97 (0.92–1.01)
Lyles, 2008 [41]	3,381	27,284	50-64 years	Ref
			65-74 years	HR = 1.10 (0.89 1.37)
			75- 84 years	HR = 1.25 (1.02 1.54)
			85+	HR = 1.38 (1.13 1.70)

RR = Relative Risk, OR = Odds Ratio, HR = Hazard Ratio, SD = standard deviation.

##### BMD

Two studies reported on the effect of BMD for predicting fractures, but used different BMD cut-off points and thus could not be pooled. Broe [35] reported a 2.76 increased fracture risk per 1 SD decrease in BMD (RR = 2.76, 95% CI: 1.69-4.61) and Chandler [38] indicated a 90% increased fracture risk for residents with a BMD below median of 0.296 g/cm^2^ (HR = 1.90, 95% CI = 1.40-2.60). Sample sizes and the number of fractures were small, and only females were included in the Chandler et al. study [38]. Low quality evidence suggests that there may be a moderate to large increased risk of fracture among residents with lower BMD. However, it is unclear whether risk of fracture is associated with a certain threshold of BMD, or if the risk increases linearly with decreasing BMD as different methods of measuring BMD were used.

##### Glucocorticoid use

One study that assessed the effects of current glucocorticoid use [36] on fracture risk suggested that it may be associated with a 5-fold increased risk of fractures (HR: 5.65, 95% CI = 1.77–18.0). The minimum dose and duration of glucocorticoid use was not reported. The large confidence intervals suggest imprecision in the estimate, and the analysis was restricted to subpopulation of patients who were ambulatory, suggesting indirect evidence. Low quality evidence suggests there may be a large increased risk of fractures among long term care residents with a history of glucocorticoid use.

##### Rheumatoid arthritis

One study found a HR: 1.08, 95% CI = 1.01-1.17 for arthritis (measured using MDS-RAI) and new fractures [41]. As information was not available to determine which individuals had rheumatoid arthritis specifically, the evidence provided is indirect. There is low quality evidence that there may be little to no effect of arthritis on fracture risk.

##### Other risk factors previously identified in FRAX

None of the included studies assessed the effects of parental fractured hip, current smoking, secondary osteoporosis and alcohol intake on fracture risk in LTC settings.

#### 2. Potential predictors not included in FRAX – applicable to frail elderly in LTC homes

##### Psychotropic medication use

Five studies explored the association of psychotropic medication use and fracture risk with a pooled effect size from three studies (46,366 participants) of RR = 1.33 (95% CI = 1.05-1.69) (Figure 2). Heterogeneity was substantial (I^2^ = 70%) and might be explained by the different types of psychotropic medication used: Colon-Emeric [40] assessed anxiolytic use, Dobnig [36] assessed opiates use, and Lyles [41] assessed antipsychotic use. The estimates of the effect of psychotropic medication use on fracture risk across the three studies were consistent and ranged from a small to large effect. One of the three studies included a selective subpopulation [36].

Two additional studies compared conventional antipsychotic medication use to atypical antipsychotic medication use. These studies could not be pooled due to the use of different comparators. The studies suggest no effect to a small increased risk but the confidence intervals are wide (RR = 1.49, 95% CI = 0.93–2.41 (Huybrechts, 2011) [39] and RR = 1.29, 95% CI = 0.95–1.76 (Huybrechts, 2012) [33]). Due to the substantial heterogeneity, imprecise results and potential indirect evidence, there is very low quality evidence and the association of psychotropic medication use on risk of fractures is uncertain.

##### Cognitive impairment

The pooled effect size from two studies (14,403 participants) investigating an association between cognitive impairment and risk of fractures was RR = 1.53, 95% CI = 1.09-2.14 (Figure 2). Heterogeneity was low (I^2^ = 30). Cognitive impairment in these two studies was measured using the cognitive performance scale within the Minimal Data Set. A third study could not be pooled as it used the mini–mental state examination (MMSE) score to measure cognitive impairment [22]. Results from that study found a 10% reduced risk of fractures per 5 units improvement in the MMSE score (OR = 0.90, 95% CI = 0.82-0.99) [22]. Moderate quality evidence from the pooled and non-pooled studies suggests that there is probably a small increase in fracture risk in patients with cognitive impairment.

##### Mobility

The three studies [36, 37, 40], reporting the association between mobility and fractures used different definitions and could not be pooled, and Nakamura [37] did not provide data to convert the effect measures from HR to RR. Results from these studies were heterogeneous. One study reported [40] OR: 1.14, 95% CI: 0.78-1.64 in residents who could independently transfer; another study reported HR: 0.99, 95% CI: 0.79–1.23 per unit increase in mobility score [36]; and another study reported HR: 2.19, 95% CI: 1.35-3.57 in ambulatory residents [37]. Due to inconsistent results, and possible indirect evidence from one study in a selective population [37], there is very low quality evidence and the effect of mobility is uncertain.

##### Falls

We pooled four studies (44,560 participants) and found a RR = 1.28, 95% CI = 1.04-1.58 (Figure 2). Heterogeneity was moderate (I^2^ = 41%). Post hoc analysis excluding Visentin [34] (that only included mobile patients in the analysis) completely explained the observed heterogeneity (I^2^ = 0) and only slightly attenuated the effect measure (RR = 1.22, 95% CI = 1.12-1.32). Effects of fall history on fracture risk across individual studies ranged from small to moderate effects. The quality of evidence was assessed as moderate for a small to moderate increased risk of fractures among fallers compared to non-fallers.

#### 3. Tools developed based on assessment of risk factors among LTC residents

Of the 13 studies included in this review, only three developed a risk prediction tool based on the risk factors identified as important for fracture risk among LTC residents. Colon-Emeric [40] and colleagues developed a predication tool based on the risk factors identified in a cohort of 28,802 residents aged 65 and older from North Carolina’s Medicare Skilled Nursing Facility. The identified risk factors varied by gender; the prediction tool for females included the age group, cognitive impairment, anxiety, anxiolytic use, and wandering training. The tool for males included different risk factors: age group, weight loss, cognitive impairment, high school education, osteoporosis, pathologic bone fracture, chronic obstructive pulmonary disease (COPD), glaucoma, and standing balance impairment.

Girman and colleagues [21] conducted an exploratory analyses on data extracted from the Minimum Data Set (MDS), a standardized data collection system utilized in nursing homes, to determine the factors associated with fractures. An algorithm was developed from these factors which included age, weight, height, locomotion on unit, a fall in the previous 180 days, activities of daily living score, cognition scale score, and urinary incontinence. In a validation sample among LTC residents, the scoring algorithm had sensitivity of 0.70 and specificity of 0.39.

Chen and colleagues [31] developed a fall related fracture risk tool after following 2,000 older adults admitted to LTC homes in Australia for two years. The tool included weight, lower leg length, balance, cognitive function, type of institution, fracture history and falls in the past year. Fracture rates estimate from the developed tool correlated well with observed fracture rates, the area under the receiver operating curve (ROC) was 0.68 (95% CI: 0.67-0.69). External validation of this tool was not conducted.

As summarized above, the three tools identified in this review indicated good predicitive properities [21, 31, 40]. However, none of the tools were validated externally. Further validation is required to ensure that similar results are replicated in a different population of patients before recommending these tools in clinical practice.

## Discussion

### Summary of evidence

We systematically reviewed the literature on the association between incident fractures and risk factors used in the FRAX assessment tool and other factors which are potentially relevant to fracture risk in LTC settings.

Overall we found low to moderate quality evidence that most of the predictors we examined may be associated with small to moderate increases in fracture risk. Specifically, we found that history of prior fracture is probably associated with a moderate increase in fracture risk, while being a woman, cognitive impairment and a history of falls are probably associated with small increases in fracture risk. These associations were all supported by moderate quality evidence. We are less confident about the effects of predictors for which there was only low quality evidence. These predictors included BMI and arthritis which may have little to no effect on fracture risk; older age which may be associated with a small increase in fracture risk; and BMD and glucocorticoid use which may be associated with moderate to large increases in fracture risk. Lastly, the effects of mobility and psychotropic medication use are still uncertain.

None of the studies reported data on several factors included in the FRAX tool, such as parental hip fracture, current smoking, secondary osteoporosis, or alcohol intake. Although these risk factors are important predictors in community dwelling adults this information may be difficult to capture [42] or document systematically for residents in LTC settings.

As with community dwelling individuals, history of a prior fracture had the strongest impact on incident fractures. Available evidence suggested that among LTC residents, the strength of the relationship between incident fractures and prior hip fractures or other (non-hip) prior fractures may be similar. Our results suggest that the effect of hip and non-hip fractures was stronger compared to any prior fractures. This finding might be due to the variation in measuring prior fractures among studies. Not all studies clarified their definition of prior fractures, although one of the two studies [22] using any prior fractures (which also had a large sample size) specified that they asked about any fractures since age 50 years old. Whereas two of the three studies categorizing prior fractures as hip and non-hip [40, 41] asked for more recent fractures, and one of these [41], specified prior fractures requiring hospitalization only. It was not possible to examine the effect of prior vertebral fractures upon subsequent fracture risk.

While age may be associated with a small increased risk of fracture and BMI associated with little or no effect, they were assessed as continuous variables in the majority of included studies. Subsequently, a cut off point for age and BMI, which would be clinically useful to identify those at high risk, could not be determined. Similarly, although cognitive impairment is probably associated with an increased fracture risk, there was insufficient information to provide the clinician with more specific information regarding the level of cognitive impairment that would best predict who is most at risk of experiencing a fracture. In addition, studies used various measures of mobility, and different types and dosages of psychotropic medications making it difficult to draw conclusions or explore the associated risks.

We also found that BMD may be associated with a moderate to large increased risk of fractures. However, the role of BMD as a criterion in LTC settings is unclear. Obtaining a BMD test for a frail resident can be challenging as they are required to be transported to another facility for the test and may find it difficult to lie flat for the scans. Furthermore, Rodondi [8] observed that measuring BMD did not modify the fracture probability obtained by the FRAX tool alone in LTC residents [8]. In Greenspan’s investigation of screening strategies using the FRAX algorithm with femoral neck BMD, 80% of residents would have been considered candidates for treatment however, 10% of those with silent vertebral fractures would have been missed [20].

Our findings, confirm that additional factors, such as psychotropic medication use, falls and cognitive impairment may be important factors to consider when assessing fracture risk among LTC residents. Further data is required to understand the importance of mobility and psychotropic medication use in LTC settings.

A number of tools included risk factors that are pertinent to LTC settings and should be acknowledged. For example, Q fracture [18] provides an estimate of fracture risk for any time between one and ten years and includes risk factors, such as dementia and falls. This tool does not rely on BMD measurements making it potentially applicable for use in LTC homes.

Another tool, the FRAMO (Fracture and Mortality Index) [43, 44] was developed to predict two-year hip and fragility fracture risk in community dwelling elderly women and may potentially be applicable in LTC settings. The FRAMO index includes age ≥ 80 years, weight <60 kg, prior fragility fracture, impaired ability to rise from a chair, a history of falls and low BMD as measured by heel ultrasound. These tools were derived in community dwelling adults and have not been validated among LTC residents. Further evaluation is required before these tools can be applied more broadly in this clinical setting.

Our review identified three studies that developed scoring algorithms for fracture risk in LTC settings that are not dependent on diagnostic imaging and include factors that are easy to measure in LTC settings [21, 31, 40]. However these tools have not been adequately validated in populations different from the ones used for developing the tools. Further validation is required to ensure that similar results are replicated in a different population of patients before recommending these tools in clinical practice.

### Quality of the evidence

Given the small number of studies assessing each risk factor identified a priori, we were not able to pool the effects across studies for some risk factors. Subgroup analyses were also not always possible and thus we were not able to explain heterogeneity. The observed heterogeneity was likely due to variations in the measurement methods used. In addition, the studies did not always clearly define the predictors used and how they were measured. Pooled studies may thus have had different definitions. For example, it was not clear how cognitive impairment was defined and the dose of the glucocorticoids and psychotropic medications was not always clear.

Risk of bias across studies was low, yet close to half of the studies in this review (five studies) were selective in their inclusion criteria, and excluded LTC residents who were males, without prior fractures, or who were not mobile. This resulted in indirect evidence and was a major reason the quality of evidence was assessed as lower.

### Limitations

This synthesis focused on a review of the evidence for risk factors included in the FRAX tool and four additional factors, which are common in LTC settings. There may be other factors associated with fracture risk that we did not address. Our search strategy was designed to be comprehensive; nevertheless, it is possible some relevant studies were missed. We also restricted the inclusion criteria to prospective cohort studies. However we believe that evidence from these studies would represent higher quality evidence compared to cross-sectional and retrospective studies.

The use of the GRADE method to evaluate the evidence in studies of disease prognosis is relatively new and was adapted for this study. While using a novel approach may be regarded as a limitation, the rigor of the GRADE system is notable.

## Conclusions

In addition to the criteria used in the FRAX assessment tool to assess risk, we found other predictors, which may be associated with a small increase in the risk of fractures in LTC settings. Factors in addition to the FRAX predictors included falls and cognitive impairment, and the effect of psychotropic medication use and mobility are still uncertain. Given that these predictors may be specific to LTC settings and that certain FRAX predictors may be hard to assess or not prevalent among LTC residents, an assessment tool which assesses those at risk of fracture in LTC settings is needed. The tool should be validated among LTC residents, and modified based on the results, before use in clinical settings.

## Appendix 1: Search strategy in Medline

1. ("nursing home*" or nursinghome).mp. (32860)
2. "care home*".tw. (1486)
3. ("residential home*" or "residential facilit*").tw. (1280)
4. Residential Facilities/(4324)
5. Homes for the Aged/(10155)
6. Long-Term Care/(20221)
7. Residential Facilities/(4324)
8. Assisted Living Facilities/(708)
9. Nursing Homes/(26216)
10. Skilled Nursing Facilities/(3443)
11. (long-term adj3 care).mp. (28746)
12. (long adj2 term adj2 care).mp. (28144)
13. or/1-12 (66641)
14. Fractures, Bone/(43616)
15. (bone adj3 fracture$).mp. (51254)
16. Hip Fractures/(9698)
17. Femoral Neck Fractures/(7027)
18. Fractures, Compression/(843)
19. Fracture$.mp. (193958)
20. or/14-19 (193958)
21. Risk Assessment/(144903)
22. Risk Factors/(481580)
23. "Predictive Value of Tests"/(119461)
24. screening.mp. (322940)
25. (risk adj3 predict$).mp. (21360)
26. (selection adj2 strategy).mp. (925)
27. risk profil$.mp. (6091)
28. risk index.mp. (1548)
29. or/21-28 (980517)
30. 13 and 20 and 29 (369)

## Authors’ information

RK is a PhDc in the Department of Clinical Epidemiology and Biostatistics and a research fellow at the Population Health Research Institute at McMaster University, Hamilton, Ontario, Canada.

NS is an assistant professor in the Department of Clinical Epidemiology and Biostatistics at McMaster University, Hamilton, Ontario, Canada.

LP is a Research Associate, Department of Medicine at McMaster University.

OO is a MSc in the Department of Public Health and Health Systems at University of Waterloo, Ontario, Canada.

LG is a Professor of Kinesiology at University of Waterloo, Waterloo, Ontario, Canada.

CS is a Research Coordinator, Geriatric Education and Research in Aging Sciences Centre, Hamilton Health Sciences Centre, Hamilton, Ontario, Canada.

AP is a Professor of Medicine, Division of Geriatric Medicine at McMaster University, Hamilton, Ontario, Canada.

## References

[1] Papaioannou A, Kennedy CC, Ioannidis G, Sawka A, Hopman WM, Pickard L, Brown JP, Josse RG, Kaiser S, Anastassiades T, Goltzman D, Papadimitropoulos M, Tenenhouse A, Prior JC, Olszynski WP, Adachi JD, CaMos Study Group (2009). **The impact of incident fractures on health-related quality of life: 5 years of data from the Canadian Multicentre Osteoporosis Study**. Osteoporos Int.

[2] Haentjens P, Magaziner J, Colon-Emeric CS, Vanderschueren D, Milisen K, Velkeniers B, Boonen S (2010). Meta-analysis: excess mortality after hip fracture among older women and men. Ann Intern Med.

[3] Nikitovic M, Wodchis WP, Krahn MD, Cadarette SM (2013). Direct health-care costs attributed to hip fractures among seniors: a matched cohort study. Osteoporos Int.

[4] Hopkins RB, Tarride JE, Leslie WD, Metge C, Lix LM, Morin S, Finlayson G, Azimaee M, Pullenayegum E, Goeree R, Adachi JD, Papaioannou A, Thabane L (2013). Estimating the excess costs for patients with incident fractures, prevalent fractures, and nonfracture osteoporosis. Osteoporos Int.

[5] Crilly RG, Tanner DA, Kloseck M, Chesworth BM (2010). Hip fractures in long-term care: is the excess explained by the age and gender distribution of the residents?. J Aging Res.

[6] Ronald LA, McGregor MJ, McGrail KM, Tate RB, Broemling AM (2008). Hospitalization rates of nursing home residents and community-dwelling seniors in British Columbia. Can J Aging.

[7] Zimmerman SI, Girman CJ, Buie VC, Chandler J, Hawkes W, Martin A, Holder L, Hebel JR, Sloane PD, Magaziner J (1999). The prevalence of osteoporosis in nursing home residents. Osteoporos Int.

[8] Rodondi A, Chevalley T, Rizzoli R (2012). Prevalence of vertebral fracture in oldest old nursing home residents. Osteoporos Int.

[9] Nevitt MC, Ettinger B, Black DM, Stone K, Jamal SA, Ensrud K, Segal M, Genant HK, Cummings SR (1998). The association of radiographically detected vertebral fractures with back pain and function: a prospective study. Ann Intern Med.

[10] Papaioannou A, Watts NB, Kendler DL, Yuen CK, Adachi JD, Ferko N (2002). Diagnosis and management of vertebral fractures in elderly adults. Am J Med.

[11] Gold DT (1996). The clinical impact of vertebral fractures: quality of life in women with osteoporosis. Bone.

[12] Murad MH, Drake MT, Mullan RJ, Mauck KF, Stuart LM, Lane MA, Abu Elnour NO, Erwin PJ, Hazem A, Puhan MA, Li T, Montori VM (2012). Clinical review. Comparative effectiveness of drug treatments to prevent fragility fractures: a systematic review and network meta-analysis. J Clin Endocrinol Metab.

[13] Watts NB, Ettinger B, LeBoff MS (2009). FRAX facts. J Bone Miner Res.

[14] Leslie WD, Berger C, Langsetmo L, Lix LM, Adachi JD, Hanley DA, Ioannidis G, Josse RG, Kovacs CS, Towheed T, Kaiser S, Olszynski WP, Prior JC, Jamal S, Kreiger N, Goltzman D, Canadian Multicentre Osteoporosis Study Research Group (2011). Construction and validation of a simplified fracture risk assessment tool for Canadian women and men: results from the CaMos and Manitoba cohorts. Osteoporos Int.

[15] Lydick E, Cook K, Turpin J, Melton M, Stine R, Byrnes C (1998). Development and validation of a simple questionnaire to facilitate identification of women likely to have low bone density. Am J Manag Care.

[16] Black DM, Steinbuch M, Palermo L, Dargent-Molina P, Lindsay R, Hoseyni MS, Johnell O (2001). An assessment tool for predicting fracture risk in postmenopausal women. Osteoporos Int.

[17] Hippisley-Cox J, Coupland C (2009). Predicting risk of osteoporotic fracture in men and women in England and Wales: prospective derivation and validation of QFractureScores. BMJ.

[18] Hippisley-Cox J, Coupland C (2012). Derivation and validation of updated QFracture algorithm to predict risk of osteoporotic fracture in primary care in the United Kingdom: prospective open cohort study. BMJ.

[19] Bravo G, Dubois MF, De Wals P, Hebert R, Messier L (2002). Relationship between regulatory status, quality of care, and three-year mortality in Canadian residential care facilities: a longitudinal study. Health Serv Res.

[20] Greenspan SL, Perera S, Nace D, Zukowski KS, Ferchak MA, Lee CJ, Nayak S, Resnick NM (2012). FRAX or fiction: determining optimal screening strategies for treatment of osteoporosis in residents in long-term care facilities. J Am Geriatr Soc.

[21] Girman CJ, Chandler JM, Zimmerman SI, Martin AR, Hawkes W, Hebel JR, Sloane PD, Magaziner J (2002). Prediction of fracture in nursing home residents. J Am Geriatr Soc.

[22] Chen JS, Sambrook PN, Simpson JM, Cameron ID, Cumming RG, Seibel MJ, Lord SR, March LM (2009). Risk factors for hip fracture among institutionalised older people. Age Ageing.

[23] Leslie WD, Lix LM, Langsetmo L, Berger C, Goltzman D, Hanley DA, Adachi JD, Johansson H, Oden A, McCloskey E, Kanis JA (2011). Construction of a FRAX(R) model for the assessment of fracture probability in Canada and implications for treatment. Osteoporos Int.

[24] Zhang J, Yu KF (1998). What's the relative risk? A method of correcting the odds ratio in cohort studies of common outcomes. JAMA.

[25] Manager R (2008). Review Manager (Rev Man) [Computer Program]. Version 5.0 edn.

[26] Higgins JPT, Green S (2009). Cochrane Handbook for Systematic Reviews of Interventions Version 5.1.0 [updated September 2011].

[27] Hayden JA, Cote P, Bombardier C (2006). Evaluation of the quality of prognosis studies in systematic reviews. Ann Intern Med.

[28] Balshem H, Helfand M, Schunemann HJ, Oxman AD, Kunz R, Brozek J, Vist GE, Falck-Ytter Y, Meerpohl J, Norris S, Guyatt GH (2011). GRADE guidelines: 3. Rating the quality of evidence. J Clin Epidemiol.

[29] Monson RR (1980). Occupational Epidemiology.

[30] Oleckno WA (2002). Essentials Epidemiology Principles and Applications.

[31] Chen JS, Sambrook PN, Simpson JM, March LM, Cumming RG, Seibel MJ, Lord SR, Cameron ID (2010). A selection strategy was developed for fracture reduction programs in frail older people. J Clin Epidemiol.

[32] Berry SD, Samelson EJ, Ngo L, Bordes M, Broe KE, Kiel DP (2008). Subsequent fracture in nursing home residents with a hip fracture: a competing risks approach. J Am Geriatr Soc.

[33] Huybrechts KF, Schneeweiss S, Gerhard T, Olfson M, Avorn J, Levin R, Lucas JA, Crystal S (2012). Comparative safety of antipsychotic medications in nursing home residents. J Am Geriatr Soc.

[34] Visentin P, Ciravegna R, Uscello L, Molaschi M, Fabris F (1995). Site-specific relative risk of fractures in the institutionalized elderly. Gerontology.

[35] Broe KE, Hannan MT, Kiely DK, Cali CM, Cupples LA, Kiel DP (2000). Predicting fractures using bone mineral density: a prospective study of long-term care residents. Osteoporos Int.

[36] Dobnig H, Piswanger-Solkner JC, Obermayer-Pietsch B, Tiran A, Strele A, Maier E, Maritschnegg P, Riedmuller G, Brueck C, Fahrleitner-Pammer A (2007). Hip and nonvertebral fracture prediction in nursing home patients: role of bone ultrasound and bone marker measurements. J Clin Endocrinol Metab.

[37] Nakamura K, Takahashi S, Oyama M, Oshiki R, Kobayashi R, Saito T, Yoshizawa Y, Tsuchiya Y (2010). Prior nonhip limb fracture predicts subsequent hip fracture in institutionalized elderly people. Osteoporos Int.

[38] Chandler JM, Zimmerman SI, Girman CJ, Martin AR, Hawkes W, Hebel JR, Sloane PD, Holder L, Magaziner J (2000). Low bone mineral density and risk of fracture in white female nursing home residents. JAMA.

[39] Huybrechts KF, Rothman KJ, Silliman RA, Brookhart MA, Schneeweiss S (2011). Risk of death and hospital admission for major medical events after initiation of psychotropic medications in older adults admitted to nursing homes. CMAJ.

[40] Colon-Emeric CS, Biggs DP, Schenck AP, Lyles KW (2003). Risk factors for hip fracture in skilled nursing facilities: who should be evaluated?. Osteoporos Int.

[41] Lyles KW, Schenck AP, Colon-Emeric CS (2008). Hip and other osteoporotic fractures increase the risk of subsequent fractures in nursing home residents. Osteoporos Int.

[42] Wall M, Lohfeld L, Giangregorio L, Ioannidis G, Kennedy CC, Moser A, Papaioannou A, Morin SN (2013). Fracture risk assessment in long-term care: a survey of long-term care physicians. BMC Geriatr.

[43] Albertsson DM, Mellstrom D, Petersson C, Eggertsen R (2007). Validation of a 4-item score predicting hip fracture and mortality risk among elderly women. Ann Fam Med.

[44] Albertsson D, Mellstrom D, Petersson C, Thulesius H, Eggertsen R (2010). Hip and fragility fracture prediction by 4-item clinical risk score and mobile heel BMD: a women cohort study. BMC Musculoskelet Disord.

[CR45] The pre-publication history for this paper can be accessed here:http://www.biomedcentral.com/1471-2318/14/130/prepub

